# Identifying regulatory driver motifs in non-small cell lung carcinoma via a systematic approach

**DOI:** 10.1371/journal.pone.0340798

**Published:** 2026-02-11

**Authors:** Rahul Kumar, Sheersh Massey, Sarah Albogami, Abdulaziz A. Aloliqi, Abdulaziz Asiri, Maher M. Aljohani, Hashim M. Aljohani, Atul Kumar, Kapil Dev

**Affiliations:** 1 Medical Biotechnology Lab, Department of Biotechnology, Jamia Millia Islamia, New Delhi, India; 2 Human Genetics Lab, Department of Biosciences, Jamia Millia Islamia, New Delhi, India; 3 Department of Biotechnology, College of Science, Taif University, Taif, Saudi Arabia; 4 Department of Basic Health Sciences, College of Applied Medical Sciences, Qassim University, Buraydah, Al-Qassim, Saudi Arabia; 5 Department of Medical Laboratory Sciences, College of Applied Medical Sciences, University of Bisha, Bisha, Saudi Arabia; 6 Department of Basic Medical Sciences, College of Medicine, Taibah University, Medina, Saudi Arabia; 7 Department of Clinical Laboratory Sciences, College of Applied Medical Sciences, Taibah University, Medina, Saudi Arabia; Apeejay Stya University, INDIA

## Abstract

**Background:**

Lung cancer exhibits highest incidence among all cancer types worldwide and even after rigorous research and advanced treatment strategies, it constitutes a primary cause of cancer-related mortality. Non-small cell lung cancer is the predominant subtype, constituting the majority of lung cancer cases. Therefore, exploring novel biomarkers is crucial for betterment of diagnostic and therapeutic approaches.

**Methods:**

The meta-analysis was performed using GEO datasets, to explore the differentially expressed genes (DEGs) and miRNAs (DEMs) in the non-small cell lung cancer (NSCLC) cases. We explored the ChEA database to extract the relevant transcription factors regulating the expression of our hub genes. Further, based on the highest degree of centrality, the feed-forward loop was identified with highest sub-network motif comprising of gene-TF-miRNA. We used pathway and GO term enrichment analysis to determine the importance of these DEGs in different biological processes.

**Results:**

In NSCLC, we found 950 differentially expressed miRNAs and 1761 genes were recognized exhibiting the significant change in expression (p < 0.05). Further, we investigated the role of sub-network motif in patient survival, hsa-miR-5010 was found to be significantly linked with patient outcome in Lung Adenocarcinoma (LUAD) (p = 0.033) and Lung Squamous Cell Carcinoma (LUSC) (p = 0.013) while SMAD4 (p < 0.001) and NRG1 (p < 0.001) expression exhibited prognostic significance in LUAD cohort only.

**Conclusion:**

Our data indicated that NRG1-SMAD4-miR-5010-5p was the most prominent sub-network motif engaged in NSCLC patients based on the degree of centrality. In vitro mechanistic studies will provide better understanding on the role of NRG1-SMAD4-miR-5010-5p motif in NSCLC cases.

## 1. Introduction

For numerous decades, lung cancer is ranked amongst the most prevalent type of cancer globally [1]. Although it stands as the third most common cancer, following breast and prostate cancer, it holds the highest proportion of cancer-related fatalities, accounting for 22% of such deaths [2]. Approximately 80% of lung cancer associated deaths can be linked to smoking. Other factors known to increase the risk of developing lung cancer include exposure to radon, prolonged and cumulative exposure to air pollution- especially emissions comprising polycyclic aromatic hydrocarbons (PAH), asbestos, as well as personal or family history of lung cancer [3,4]. According to the World Health Organization (WHO), lung tumors are categorized into two main types: non-small cell lung cancer (NSCLC), which makes up 80–85% of all cases, and small cell lung cancer (SCLC), accounting for the remaining 15% [1,5].

Compelling evidence supports the significant connections of long non-coding RNAs (lncRNAs) in several diseases, with a particular emphasis on cancer, including NSCLCs. Recent research has explored the expression patterns of lncRNAs in NSCLCs. MicroRNAs (miRNAs), a class of small non-coding RNAs, play vital roles in post-transcriptional regulation of gene. Dysregulation of miRNA expression has been implicated in several cancer types, including NSCLC, where they can function both as tumor suppressors and oncogenes [6,7]. Xu et al. found 2420 lncRNAs with differential expression (fold change ≽2) between lung adenocarcinoma (LAD) and normal tissue (NT) samples using high-throughput microarrays. Out of these, 1213 lncRNAs showed upregulation, while the remaining 1207 lncRNAs demonstrated downregulation [8,9]. Gaining insights into the complex interactions involving miRNAs, messenger RNAs (mRNAs), and transcription factors (TFs) is crucial for unravelling the molecular mechanisms of NSCLC, providing potential avenues for diagnosis and therapy.

In the last few years, the domain of bioinformatics has witnessed the emergence of potent tools for unravelling intricate regulatory networks within cancer cells. The integrated examination of high-throughput data encompassing miRNA and mRNA expression, alongside the prediction of regulatory motifs involving miRNAs, target mRNAs, and related transcription factors (TFs), has yielded fresh perspectives on the regulatory setting of non-small cell lung carcinoma (NSCLC) [10]. Harnessing computational approaches enables the identification of pivotal regulatory modules governing the progression of NSCLC, thereby aiding in the design of targeted interventions [11].

In this investigation, we conducted a thorough bioinformatics examination of miRNA and mRNA expression patterns in NSCLC with the goal of unveiling regulatory motifs that govern gene networks associated with cancer. Our systematic computational analysis includes gene and miRNA expression curation followed by their validation using publicly available datasets. Also, evaluating their significance in patient outcome via survival analysis and finally identifying novel regulatory motif involving miRNA, genes, and transcription factors (TFs), presenting itself as a promising candidate for potential therapeutic targeting in NSCLC. The insights derived from our study illuminate the complex regulatory mechanisms that underlie NSCLC and establish a basis for subsequent experimental validation and exploration of therapeutic avenues.

## 2. Materials and methods

**Work Flow Chart**


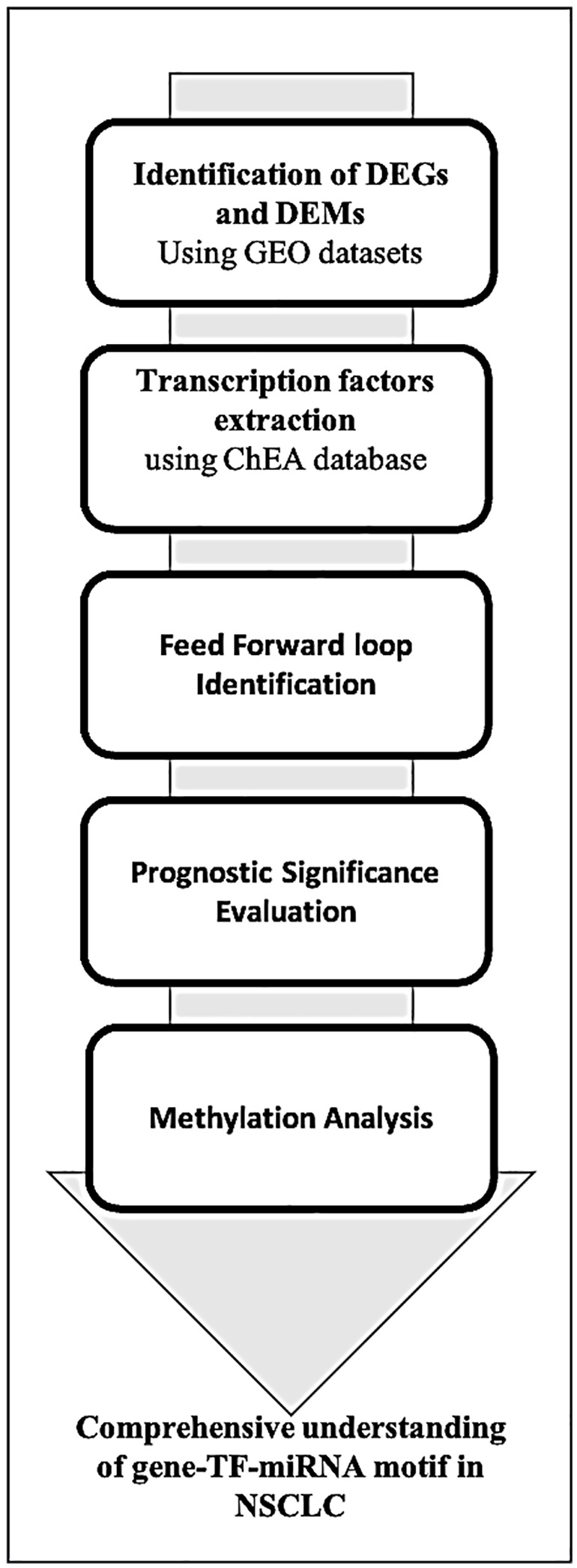


### 2.1. Procurement of Microarray data and preliminary processing

We obtained miRNA and mRNA expression profiles from publicly available datasets related to Non-Small Cell Lung Cancer (NSCLC) from the Genome Expression Omnibus (GEO) database [12]. We refined our search by applying specific inclusion criteria: (1) we considered only samples from the ‘Homo sapiens’ species and of the ‘expression profiling by array’ type; (2) we selected datasets that included both processed and raw microarray data; (3) we focused on datasets with sample sizes ranging from 20 to 1000. The miRNA and mRNA expression datasets were acquired using the accession numbers GSE53882 and GSE31799, respectively. We downloaded the series matrix (processed) files for both datasets. Additionally, as the data was pre-normalized, preprocessing steps involved background correction and removal of duplicates.

### 2.2. Identification of DEGs and DEMs

We employed the R statistical software (https://www.r-project.org/), specifically employing the Limma package [13], to conduct an analysis of the expression profiles obtained from the selected datasets. This package facilitated the implementation of the t-test for assessing significance, resulting in corresponding p-values. Subsequently, miRNAs and genes exhibiting p-values less than 0.05 and fold changes within the range of 0.5 to 1.0 were identified and classified as differentially expressed miRNAs (DEMs) and differentially expressed genes (DEGs), respectively [14,15].

### 2.3. Enrichment analysis of pathways and Gene Ontology (GO) terms

We gathered information on the top 10 significantly enriched pathways and Gene Ontology (GO) terms (p-value < 0.05) associated with the identified DEGs using the Enrichr web-based tool [16]. Our selection included the Reactome pathway database, as well as the GO Biological Process (GO-BP), GO Molecular Function (GO-MF), and GO Cellular Component (GO-CC) libraries for compiling the pathway and GO term data.

### 2.4. Analysis and construction of 3-node miRNA FFL

Relevant transcription factors (TFs) with a significance score (p-value) less than 0.05, which regulate our central mRNAs, were obtained from the ChEA v3.0 database as it offers experimentally validated TF-mRNA interactions [17]. Subsequently, the corresponding miRNAs (score greater than 0.95 and binding exclusively to the 3′ UTR region) that suppress our central mRNAs, along with the significant TFs obtained from ChEA, were retrieved from cross-validated comprehensive list of miRNAs in miRWalk v3.0 database [18]. To design a 3-node miRNA Feed-Forward Loop (FFL), only the common miRNAs that repress both our mRNAs and transcription factors were maintained. Ultimately, all three interaction pairs “miRNA-mRNA, TF-mRNA, and mRNA-TF”—were calibrated according to shared miRNAs and definitive TFs. The pairings were subsequently combined to create a 3-node miRNA feedforward loop, and the network was analysed using Cytoscape (http://www.cytoscape.org/) [19].

### 2.5. Survival analysis

We performed survival analysis utilising the Kaplan-Meier Plotter to assess the prognostic importance of hsa-miR-5010, SMAD4, and NRG1(https://kmplot.com/analysis/) [20], a publicly accessible database enables the gene and miRNA expression on clinical outcomes across diverse cancer types. Following the selection of the specific miRNA or gene of interest, our focus was directed towards lung adenocarcinoma and lung squamous cell carcinoma. To ensure data accuracy, we applied two essential filters: ‘Invert HR values below 1’ to highlight adverse survival associations and ‘Compute median over the entire dataset’ to obtain robust estimates.

### 2.6. MethSurv analysis

The predictive relevance of CpG methylation in LUAD and LUSC cohorts was assessed using the web-based program MethSurv (https://biit.cs.ut.ee/methsurv). This tool is useful for survival analysis based on individual CpG sites using TCGA data. CpG sites of SMAD4 and NRG1 were analyzed to determine their association with patient overall survival. The results provided insights into potential methylation-based prognostic biomarkers in NSCLC.

## 3. Results

### 3.1. Acquisition of microarray data and pre-processing

Under the defined search conditions in the methods, we acquired 36 miRNA and 66 mRNA expression profiles. We specifically chose datasets with accession numbers GSE53882 (miRNA, including tumor and control samples) and GSE31799 (mRNA, including Adenocarcinoma and Squamous cell carcinoma samples). The miRNA dataset comprised 397 samples from patients diagnosed with tumors and 151 corresponding adjacent non-cancerous tissues. Conversely, the mRNA expression data consisted of 31 samples with adenocarcinoma and 20 samples with Squamous cell carcinoma.

We enhanced dataset reliability through vital pre-processing steps, including normalization and probe ID mapping using GEO2R. This yielded accurate quantile normalized and RMA normalized expression values for GSE53882 and GSE31799, respectively, tied to probes. The resulting tab-delimited text file after batch correction using PCA-based method formed the basis for robust analyses.

### 3.2. Identification of DEGs and DEMs

Upon implementing the specified thresholds, we identified a total of 950 differentially expressed miRNAs (DEMs) and 1761 genes (DEGs) in relation to their respective varying conditions. By incorporating criteria accommodating low-fold changes, we ensured a comprehensive assessment of tumor vs. control group variations, maximizing DEMs and DEGs detection.

**Fig 1:**
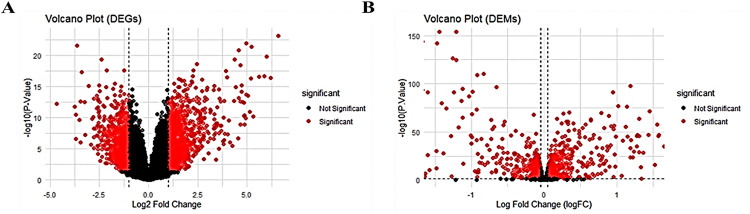
Volcano plot representing DEGs and DEMs: Log2 Fold Change, Y-axis: -Log10 (P-Value). Red dots significant and black dots are insignificant. (**A)** DEGs. **(B)** DEMs.

**Fig 2:**
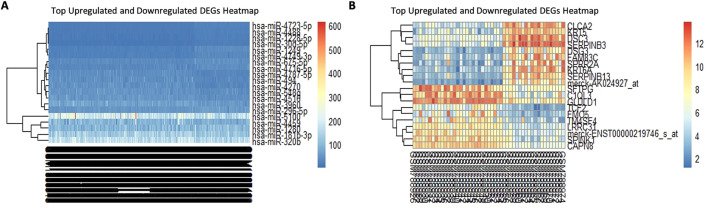
Heatmap illustrating. (A) Top 10 up and downregulated DEMs. Gene’s positions are indicated by vertical multicolour bands in the right panel. (B) Top 10 up and downregulated DEGs. Gene’s positions are highlighted by vertical multicolour bands in the right panel.

Table 1 details the compiled upregulated and downregulated DEMs and DEGs, while Fig 1A, 1B showcases volcano plots, illustrating significant vs. nonsignificant miRNAs and genes. Additionally, Fig 2A, 2B presents heatmap plots for the top 10 down and upregulated DEMs and DEGs, providing insights into their expression patterns.

**Table 1:** Upregulated and downregulated DEMs and DEGs.

GEO accession	Number of upregulated	Number of Downregulated
GSE53882(miRNAs)	516	434
GSE31799(mRNAs)	1012	749

### 3.3. Pathway and GO term enrichment analyses

**Fig 3:**
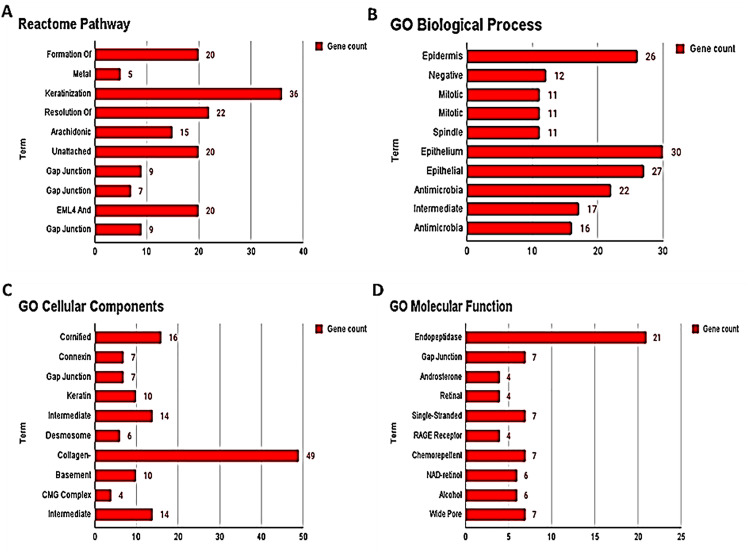
Pathway and GO term enrichment analyses. (**A)** Reactome Pathway Analysis **(B)** GO Biological Process Analysis **(C):** GO Cellular Compartment Analysis **(D)** GO Molecular Function Analysis.

We conducted a comprehensive Reactome pathway analysis using the Enrichr tool, which unveiled the participation of 88 differentially expressed genes (DEGs) in the top 10 Reactome pathways (p < 0.05). Among these, the pathway with the highest gene count was identified as “Keratinization.” Particularly noteworthy was the identification of the “Formation of Cornified Envelope” pathway as the most significant, indicating its pronounced impact in the biological context. The graphical representation in Fig 3A visually presents the top 10 Reactome pathways.

Our analysis further extended to the exploration of gene expression differences, with a combined total of 96, 66, and 85 DEGs exhibiting distinct expression patterns and engaging in the foremost 10 significant terms across the “Biological Process (BP) (p<0.05), Cellular Compartment (CC) (p<0.05), and Molecular Function (MF) (p<0.05)”. In Fig 3B, we provide a visual depiction of the distribution of noteworthy Gene Ontology (GO) Biological Process terms, demonstrating the gene count (x-axis) for each term (y-axis). Particularly notable was the prominence of “Epithelium Development,” with a comprehensive gene count of 30.

### 3.4. 3-node miRNA FFL construction and analysis

**Fig 4:**
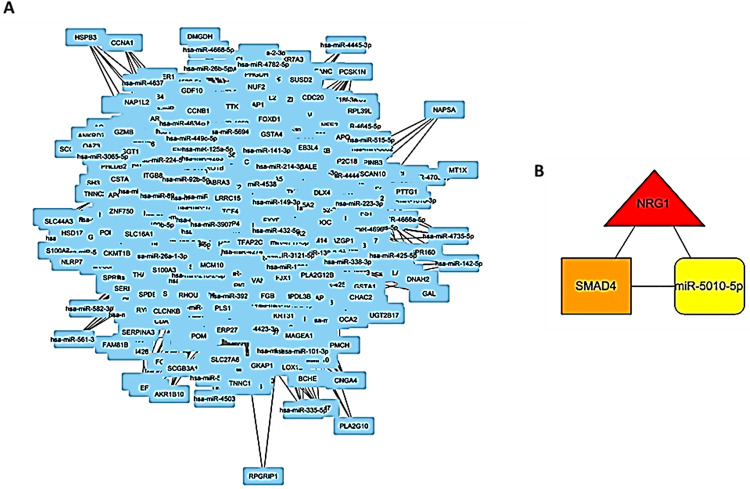
Interaction network of a miRNA feed-forward loop. (A) 3-node miRNA feed-forward loop (FFL) network Fig (B) The visual representation of the regulatory motif NRG1-SMAD4-miR-5010-5p.

The 3-node miRNA feedforward loop, seen in Fig 4A, consisted of 1871 nodes and 116,395 edges. The TF-mRNA pair comprised 9,051 edges, while the miRNA-TF and miRNA-mRNA couples accounted for 16,824 and 90,520 edges, respectively. Of the total nodes, 142 were transcription factors (TFs), 921 were messenger RNAs (mRNAs), and 808 were microRNAs (miRNAs). Utilising the Degree centrality metric, we identified the predominant subnetwork motif consisting of one transcription factor (SMAD4), one mRNA (NRG1), and one microRNA (miR-5010-5p), as illustrated in Fig 4B.

### 3.5. Survival analysis

**Fig 5:**
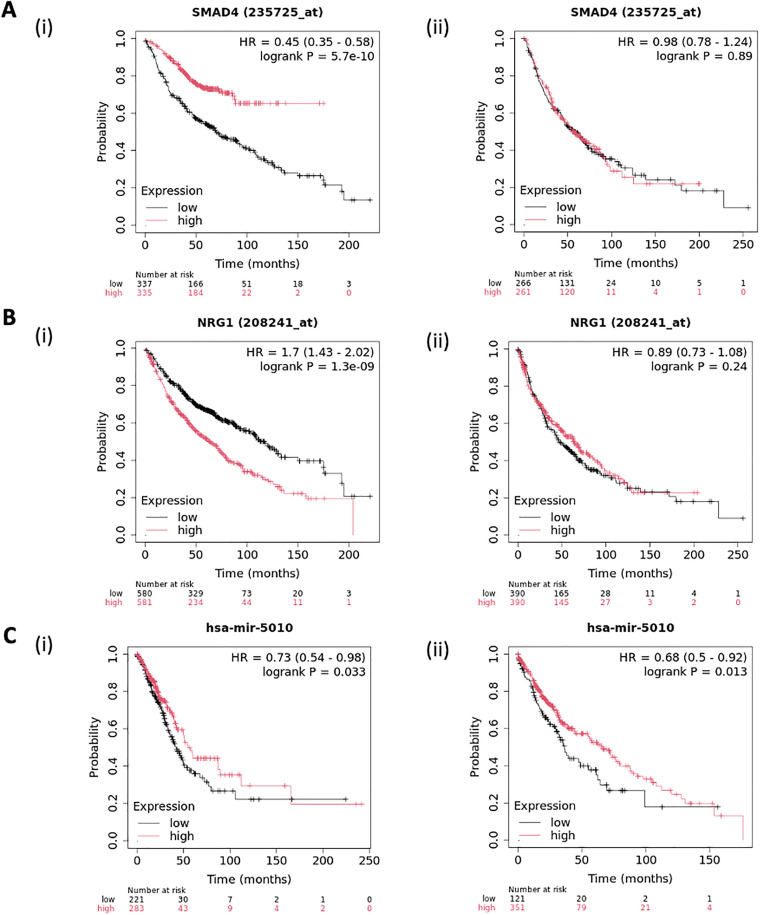
Kaplan-Meier (KM) plots showing high and low expression groups for survival analysis. **(A) (i)** SMAD4 in LUAD **(ii)** SMAD4 in LUSC **(B) (i)** NRG1 in LUAD **(ii)** NRG1 in LUSC **(C)** (i) hsa-miR-5010 in LUAD (ii) hsa-miR-5010 in LUSC.

Our survival analysis revealed the prognostic insights regarding SMAD4, NRG1 and hsa-miR-5010 in lung adenocarcinoma and lung squamous cell carcinoma. Patients with NRG1 overexpression (p < 0.001) and downregulation of tumor suppressor SMAD4 (p < 0.001) as expected correlate significantly with poor patient survival in LUAD cohort. While lower hsa-miR-5010 levels were linked to significantly shorter median survival (p < 0.05) in both LUAD (p = 0.033) as well as LUSC (p = 0.013) cohorts. These results suggest NRG1 and SMAD4 as potential prognostic markers in lung adenocarcinoma cases whereas, hsa-miR-5010 is prognostically significant in both the subtypes (Fig 5).

### 3.6. Promoter methylation analysis of SMAD4 and NRG1

**Fig 6:**
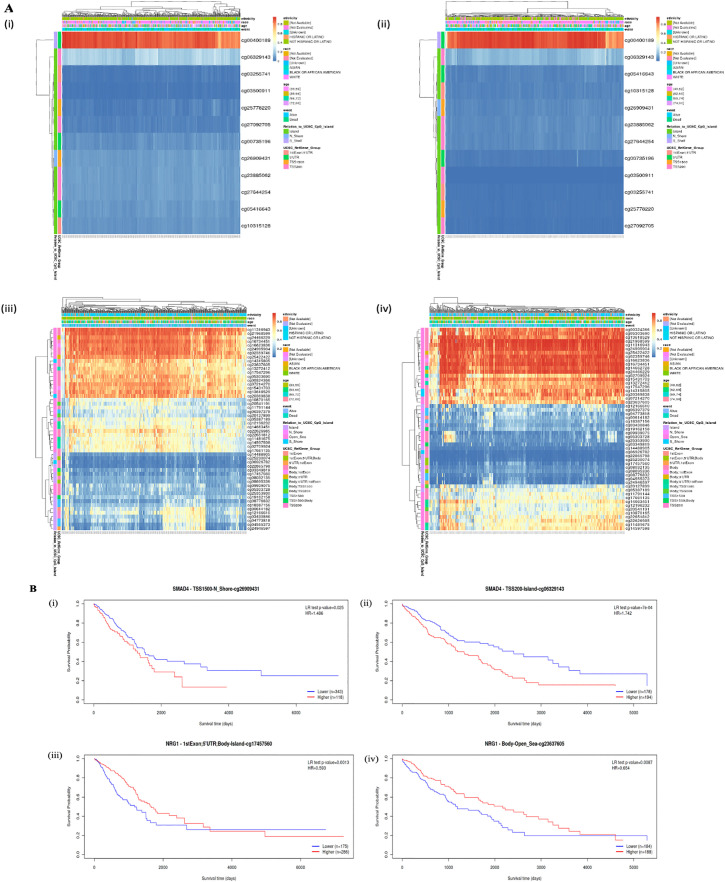
Promoter methylation analysis. **(A)** Heat map showing methylation across CpG of **(i)** SMAD4 in LUAD **(ii)** SMAD4 in LUSC **(iii)** NRG1 in LUAD **(iv)** NRG1 in LUSC. **(B)** Kaplan Meier plot for prognostically most significant CpG island **(i)** SMAD4 in LUAD **(ii)** SMAD4 in LUSC **(iii)** NRG1 in LUAD **(iv)** NRG1 in LUSC.

The promoter methylation status of various CpG islands of NRG1 and SMAD4 were analyzed (Fig 6). Heatmaps demonstrating the promoter methylation pattern across the various CpG islands throughout the LUAD and LUSC cohorts were produced. Further, these CpG islands were then investigated for prognostic significance to analyze their role in carcinogenesis and patient survival. SMAD4 CpG islands cg26909431and cgo6329143 while NRG1 CpG islands cg17457560 and cg23637605 were identified to be prognostically most significant in LUAD and LUSC respectively.

## 4. Discussion

Over the decades, the treatment strategies for non-small cell lung carcinoma have become more inclined towards a biomarker-driven personalized approach in addition to the existing therapies [21]. Molecular analysis is currently a potent tool for more efficient diagnosis and management of disease. In the current era, bioinformatics has emerged to be a promising platform for computer-aided biomarker discovery and provides the advantage of processing big data for biomarker screening. Identification and analysis of the biological molecules at the different omics levels can be efficient tools for early diagnosis and better prognosis of disease [22,23]. In this study, by using suitable inclusion and exclusion criteria a dataset from GEO having microarray profiles from two varying samples was selected. We identified DEGs from the dataset and overall, 1761 genes were discovered to exhibit differential expression, of which 1012 were upregulated and 749 were downregulated.

The Enrichr tool was used and the identified DEGs were further subjected to Reactome pathway analysis considering p value <0.05 to be significant. We identified a strong association between 88 DEGs in the top 10 Reactome pathways, with “Keratinization” exhibiting the greatest gene count. Furthermore, it was discovered that the “Formation of Cornified Envelope” was the most important pathway, demonstrating its substantial effect in the biological context. The functional significance of the identified DEGs was then evaluated using the GO enrichment tool and 96 DEGs displayed significant association across Biological Processes (BP), of which the highest gene count was observed in epithelium development. Similarly, 66 and 88 DEGs were found to have a strong connection with Cellular Compartment (CC) and Molecular Function (MF) respectively.

Epigenetic regulation plays a crucial role in controlling the gene expression [24]. Among various epigenetic regulators, miRNAs are known for their vital regulatory role in various malignancies [25,26]. In this context, we identified the differentially expressed miRNA with p-values less than 0.05 and categorized them as DEMs. Overall, 950 DEMs were identified in our study of which 516 were found to be upregulated whereas 434 were significantly downregulated. Moreover, to explore the role of transcription factors (TFs) in controlling the gene expression along with the DEMs, the ChEA database was used to extract the TFs having regulatory functions and significant association (p < 0.05) with our hub mRNA. To explore the connection among our hub mRNA, DEMs, and TFs, a feed-forward loop was constructed with the mRNA, miRNA, and TFs that were common in all three interactions i.e. miRNAs-mRNA, TFs-mRNAs, and miRNAs-TFs.

NRG1 (mRNA), SMAD4 (TF), and miR-5010-5p (miRNA) were identified to be the highest sub network motif with the highest degree of centrality measure. Neuregulin 1 (NRG1) belongs to the family of epidermal growth factors. The NRG1 expression has been reported in various malignancies like breast, lung, and prostate cancer. It is also detected to have a critical role in the prognosis of the disease and resistance to therapeutic drugs like trastuzumab [27–29]. Also, NRG1 fusion with other oncogenic genes due to chromosomal rearrangements can consequently form chimeric proteins resulting in activation of downstream pathways like PI3K-AKT. Moreover, oncogenic variants of NRG1 promote proliferation and metastasis via activating MAPK or NF-κB/MMP9 pathway and can be responsible for resistance to targeted therapies like EGFR and HER2 [30,31]. In NSCLC patients, NRG1 was found to be upregulated and its elevated expression is associated with poor survival of the patients [32]. The SMAD4 and NRG1 fusion have been reported in lung cancer cases [33]. SMAD4 is a known transcription factor located on chromosome 18q21 region and is well known to be associated with TGFβ signalling [34,35]. SMAD4 mediated TGFβ signalling plays vital role in maintenance of tissue homeostasis while is downregulation is responsible for cell proliferation and tumorigenesis. Moreover, the downregulation of SMAD4 can lead to activation of oncogenic pathways like ErbB2 and Akt leading to uncontrolled cellular growth and invasion [36,37]. Previously published literature revealed the tumor suppressive role of SMAD4 in various cancers including NSCLC, and is reported to be downregulated in cancer [38,39]. Our findings corroborated with the earlier researches and survival analysis revealed significant association of SMAD4 downregulation with good overall survival in LUAD cohort [40]. Further, miR-5010 was found to be overexpressed in the selected dataset for our study and its expression is associated with the poor survival in the non-small cell lung cancer patient. Similar role of miR-5010 is reported in gastric cancer where its expression is reported to be elevated [41]. It is important to clarify that miR-5010-5p and miR-5010 refer to the same miRNA entity; miR-5010-5p corresponds to the mature 5′ arm product derived from the miR-5010 precursor. our survival analysis revealed. In our FFL analysis, NRG1-SMAD4-miR-5010-5p is the novel axis to be associated with the NSCLC [30,36]. Further methylation analysis was done using MethSurv tool to evaluate the promoter methylation landscape of NRG1 and SMAD4. Promoter methylation status of SMAD4 CpG islands cg26909431and cgo6329143 and of NRG1 CpG islands cg17457560 and cg23637605 was observed to have most significant role in patients’ survival in LUAD and LUSC cohorts. Promoter methylation plays critical role in tumor initiation, maintenance as well as progression [42]. Promoter hypermethylation of SMAD4 gene is observed in prostate and gastric carcinoma [43,44]. Similarly, epigenetic dysregulation of NRG1 is frequently reported in cervical and breast cancer tissue [45,46]. The studies suggests the association of NRG1 expression and SMAD 4 downregulation as key players in activation of oncogenic pathways like PI3K-AKT. Also, their dysregulation is linked with disruption of cellular homeostasis and cancer progression [30,36].

The gene expression regulatory motifs plays decisive roles in carcinogenesis and extensive studies are being done to identify such motifs. For instance MYC/RB1/miR-106a exhibits oncogenic role by suppressing tumor suppressor RB1 gene at transcriptional; as well as translational levels in solid tumors. Similarly, the association of p53 and miR-34a with CDK4 have been reported in regulation of cell cycle arrest and proliferation [47–49]. Earlier studies have revealed the association of NRG1 in NSCLC; however, its regulatory interactions have not been explored. Hereby, our findings suggest the significant role of NRG1 and its regulatory partners SMAD4 and miR-5010-5p in NSCLC which could be pioneer for further studies exploring the role of respective motif in lung carcinogenesis. This study considers the publicly available GEO datasets separately for mRNA and miRNA therefore can further be validated using experimental and clinical studies. This will provide better insight into our understanding and will highlight the diagnostic and therapeutic significance of the NRG1-SMAD4-miR-5010-5p axis.

## Supporting information

S1 File Downregulated-miR. (TXT)

S2 File Upregulated-miR. (TXT)

S3 File Downregulated-mRNA. (TXT)

S4 File Upregulated-mRNA. (TXT)

S5 File miRNA_TF. (CSV)

S6 File TF_Genes. (CSV)

## Abbreviations

NSCLC: Non-small cell lung cancer
DEGs: Differentially expressed genes
DEMs: Differentially expressed miRNA
TF: Transcription factor
WHO: World Health Organization
lncRNA: long non-coding RNAs
GEO database: Genome Expression Omnibus database
GO: Gene Ontology
FFL: Feed-Forward Loop
NRG1: Neuregulin 1
TCGA: The Cancer Genome Atlas
LUAD: Lung Adenocarcinoma
LUSC: Lung Squamous Cell Carcinoma
RB1: Retinoblastoma 1
